# Identifying the factors influencing long-term care utilization by older adults in China: machine learning analysis

**DOI:** 10.1186/s12877-026-07652-y

**Published:** 2026-05-20

**Authors:** Tengyu Wang, Fei Liang, Minwen Gu, Jingyao Sun, Xin Wang, Youqi Guo

**Affiliations:** 1 The First Clinical College, China Medical University, Shenyang, People’s Republic of China; 2 Department of Breast Surgery (Surgical Oncology), The Second Affiliated Hospital, Zhejiang University School of Medicine, Hangzhou, People’s Republic of China; 3 Department of Histology and Embryology, College of Basic Medicine, China Medical University, Shenyang, People’s Republic of China; 4 CMU Center for Health Development Research, School of Health Management, China Medical University, No.77 Puhe Road, Shenyang North New Area, Shenyang, Liaoning Province People’s Republic of China; 5 College of Marxism, China Medical University, No.77 Puhe Road, Shenyang North New Area, Shenyang, Liaoning Province 110122 People’s Republic of China

**Keywords:** Older adults, Long-term care, Machine learning

## Abstract

**Background:**

To address the aging population in China, local governments began to encourage the establishment of formal care services and supplement informal care in 2015, thereby increasing the availability of different types of long-term care (LTC). In this study, nationwide survey data were accessed to identify factors influencing the types of LTC utilization by older adults in China.

**Methods:**

Data from 2,305 eligible older adults receiving care were retrieved from the 2020 China Health and Retirement Longitudinal Survey. The independent variables were structured around the predisposing, enabling, and need factors of the Andersen healthcare utilization model, while the dependent variable was categorized by the type of long-term care utilized (informal versus formal care). The analytical pipeline included a 70/30 train-test data split, Bayesian Ridge multiple imputation for missing values, and LASSO logistic regression for feature selection. To correct for severe classification imbalance, cost-sensitive learning via algorithmic class weighting was applied. Eleven machine learning (ML) models were constructed and optimized using fivefold cross-validation within the training set. Finally, the SHapley Additive exPlanations (SHAP) interpretability framework was used on the independent test set to evaluate variable importance, dependencies, and interactions.

**Results:**

Descriptive chi-square test results indicated that age, marital status, social activity, smoking, income state, residence, and living arrangement significantly the type of care received. Following feature selection and evaluation across the 11 ML models, the Random Forest model achieved the highest predictive performance on the independent test set. Subsequent SHAP interpretation of the Random Forest model identified marital status, living arrangement, social activity, physical activity, age, and residence as the most associated variables for formal care utilization. Analysis of specific impacts showed that individuals who lived alone, were over the age of 80, participated in weekly physical activities, or did not participate in social activities were more inclined to use formal care. Overall, marital status, living arrangement, and social activity participation were identified as the key interacting factors associated with the types of long-term care utilized by older adults in China.

**Conclusions:**

Living arrangement, social activity and residence were the most significant factors associated with the types of LTC utilization by older adults in China. Overall, enabling and predisposing factors had a greater influence than the need factors. These findings not only demonstrate the potential value of ML for LTC policy development, but also provide empirical support for the Chinese government to adopt targeted interventions that enhance LTC service accessibility and affordability.

**Supplementary Information:**

The online version contains supplementary material available at 10.1186/s12877-026-07652-y.

## Background

Due to the significantly increased life expectancy and sharply decreased birth rate, China is currently challenged with an aging population. According to the National Bureau of Statistics of China, the population aged 60 and above in China was 296.97 million, accounting for 21.1% of the total population 2023 [1]. By 2040, the number of people aged 60 and above in China is expected to reach 402 million, accounting for 28% of the population [2]. In response to this demographic challenge, the Chinese government has implemented a national strategy to actively address population aging.

The greater incidence of illness of an aging population has led to an increased demand for long-term care (LTC). The World Health Organization stated the LTC was an important strategy to promote “healthy aging” and national LTC systems should be established [3]. In response to aging populations, many high-income countries have established comprehensive formal and informal LTC systems. Generally, informal care is defined as the care voluntarily given by family and friends, and formal care is defined as the care provided by trained professionals paid from public or private providers [4] [5]. In recent years, these countries have been continuously improving their LTC systems to meet the needs of older adults and their families. Consequently, there has been growing scholarly interest in examining the factors associated with variations in LTC utilization patterns. Numerous studies have indicated that the differential utilization of informal and formal care is influenced by multiple factors, including: education level [6], gender and income [7, 8], social networks [9], availability of LTC services [10], insurance [11], type of illness [12, 13, 14, 15], frailty [16].

By contrast, the Chinese government currently does not generally provide formal LTC programs or assistance to informal caregivers. Therefore, relatively few studies have investigated utilization of LTC systems by older adults in China. A prior study of daily assistance and LTC services suggested that the amount of social security benefits, including pension and medical insurance, have a great impact on the availability of LTC [17]. However, as an influencing factor, income had a great impact on satisfaction with LTC, as older adults from low-income families tended to be less satisfied [18]. Another study suggested that subjective perception of the needs of older adults influenced utilization of LTC services [19].

Filial piety is customary in China, thus LTC for older adults is often provided by family members. However, care provided by family members is unstable, so differences in care intensity and influencing factors have attracted much interest of researchers. The intensity of care reportedly decreases with an increase in duration and is typically maintained only for older adults with low care needs [20]. Also, the intensity of care is influenced by family income and this inequity is exacerbated by the degree of functional loss of older adults [21]. In addition, being female, living in urban areas, and having more daughters were positively associated with care intensity [22]. Meanwhile, unpaid and substantial informal care can place great burdens on family members, as caregivers in China are usually women, who experience limited wealth accumulation and lower subjective well-being [23].

In order to alleviate the plight of family caregivers, address the low birthrate, and improve happiness of older adults, the Chinese government began to encourage the establishment of formal care services to supplement informal care in 2015. These efforts include improving the nursing quality of nursing homes, training professional caregivers for older adults, allowing hospitals to provide LTC services, and increasing community home care services [24, 25]. To determine the effects of comprehensive LTC service systems in China, the latest data of the China Health and Retirement Longitudinal Study (CHARLS 2020) were analyzed to: (1) describe the types of LTC available to older adults; (2) identify the main factors associated with the types of LTC utilization by older adults; (3) provide suggestions to establish a comprehensive, affordable, and high-quality LTC services system.

## Methods

### Data source and participants

The data for this study were obtained from the CHARLS 2020 [26], which was conducted to analyze and promote interdisciplinary research on the aging population in China. The survey subjects were Chinese middle-aged and older adults aged ≥ 45 years. The dataset was comprised of personal information, family information, and indicators of health, cognition, and other variables. The survey was initiated in 2011 and continued in 2013, 2015, 2018, and 2020 in 28 provinces (including autonomous regions and municipalities), across 150 counties, and 450 communities (villages). The survey cohort consisted of about 19,000 individuals from 12,400 households. As the CHARLS 2020 data are openly available to researchers, ethical approval was not required for this study. The CHARLS 2020 database was accessed using the account 2021352609@cmu.edu.cn. 

This study enrolled the adults aged ≥ 60 years. Eligibility was determined through a two-stage assessment: First, the participants were required to answer whether they have difficulties with basic activities of daily living (ADL, i.e., dressing, bathing, eating, standing, and toilet use) and instrumental activities of daily living (IADL, i.e., household chores, cooking, shopping, phone communication, taking medications, and managing money). Individuals who experienced difficulties or unable to complete tasks were selected. After excluding missing data for age and gender, 2719 individuals were included in the database. Second, From an initial pool of 2,719 eligible participants, we further excluded 414 individuals who reported not receiving care services. Finally, 2305 individuals who received care were included for analysis.

### Construction of analysis matrix

**Fig. 1:**
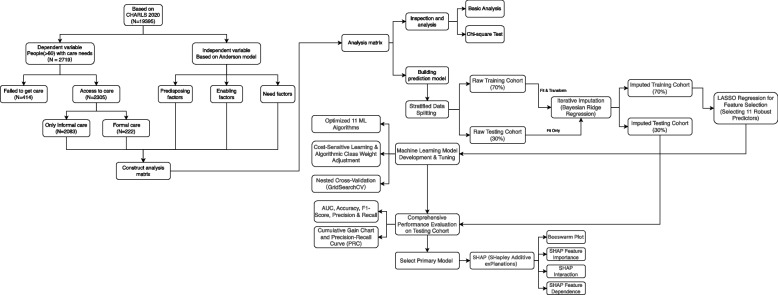
Paper Working Flow

The working flow was shown in Fig. 1. The whole work can be divided into three rigorously structured parts in compliance with TRIPOD-AI guidelines [PMID: 38636956]: (1) Independent data splitting and pre-processing: extraction of variables, followed by a strict 70/30 train-test split, MICE imputation, and baseline statistical analysis. (2) Feature selection and ML model construction: LASSO feature selection, algorithmic class weighting for imbalance, hyperparameter optimization, and fivefold cross-validation to determine the final model. (3) Clinical utility evaluation and SHAP interpretation: model evaluation on the test set and application of SHAP to resolve key factors (variable importance, dependency, and interactions).

### Dependent variables

In this study, the types of LTC utilization were regarded as observed (dependent) variables. The types of LTC utilization were selected as a dichotomous variable. In China, informal care is mainly provided by family members, while formal care is mainly provided by employers or professionals. Therefore, older adults who received care were categorized into two groups: those who only received informal care were assigned to the informal care group (*n* = 2083) and those who only received formal care or both formal and informal care were assigned to the formal care group (n = 222).

### Independent variables

The control (independent) variables were established in accordance with the three dimensions of the Andersen healthcare utilization model [27]. This represents a well-established conceptual framework widely applied in health services research, including studies on LTC utilization. The model provides a relatively complete and illustrative system composed of (1) predisposing factors that influence LTC utilization, (2) enabling factors related to necessary resources, and (3) “need” factors associated with LTC [14]. The independent variables in the three dimensions of this study include:

- Predisposing factors: (1) Gender: male/female; (2) Age: 60–69/70–79/≥ 80 years; (3) Education: below primary school (including private school)/primary school/junior high school/senior high school and above; (4) Marriage: yes/no; (5) Social activity: yes/no; (6) Smoking: yes/never; (7) Drinking: more than once a month/less than once a month/never; (8) Physical activity: yes/no; and (9) Internet use: yes/no.
- Enabling factors: (1) Insurance: yes/no; (2) Income source: industry/agriculture/both; (3) Income: < 16,000/16000–40000/> 40,000 Chinese yuan; (4) Residence: city or town center/urban and rural areas or town and township combined areas/rural areas; (5) Living arrangement: with spouse and other family members/with family members without spouse/with spouse only/alone.
- Need factors: (1) Chronic disease: yes/no; (2) Function: ADL&IADL limitations/ADL limitations/IADL limitations; (3) Health satisfaction: no/somewhat/yes; (4) Depression: yes/no.

### Data pre-processing and missing data

The raw variables from the CHARLS 2020 dataset were reconstructed to be more conducive with Andersen healthcare utilization model. Meanwhile, low-frequency variables may affect the accuracy of the ML model, so these variables were combined. These processes are explained in Supplemental material 1.

The basic information of the study participants are provided in Table 1. Data analysis as conducted with the R package “tableone” (0.13.2). Analysis of the control variables was based on the classification of the observed variables. The chi-square test was performed. *P* value < 0.05 was considered statistically significant.

**Table 1:** The characteristics of the study population

	level	Overall	Informal Care	Formal Care	*P* value
n		2305	2083	222	
Gender (%)	Male	877 (38.0)	794 (38.1)	83 (37.4)	0.888
	Female	1428 (62.0)	1289 (61.9)	139 (62.6)	
Age (%)	60–69	992 (43.0)	918 (44.1)	74 (33.3)	< 0.001
	70–79	829 (36.0)	753 (36.1)	76 (34.2)	
	> = 80	484 (21.0)	412 (19.8)	72 (32.4)	
Education (%)	Below primary school (including private school)	87 (55.1)	76 (53.9)	11 (64.7)	0.616
	Primary school	24 (15.2)	23 (16.3)	1 (5.9)	
	Junior high school	25 (15.8)	23 (16.3)	2 (11.8)	
	Senior high school and above	22 (13.9)	19 (13.5)	3 (17.6)	
Marriage (%)	Yes	1575 (68.3)	1478 (71.0)	97 (43.7)	< 0.001
	No	730 (31.7)	605 (29.0)	125 (56.3)	
Social Activity (%)	Yes	752 (32.6)	654 (31.4)	98 (44.1)	< 0.001
	No	1553 (67.4)	1429 (68.6)	124 (55.9)	
Smoking (%)	Have a smoking habit	826 (89.6)	758 (90.6)	68 (80.0)	0.004
	Never	96 (10.4)	79 (9.4)	17 (20.0)	
Drinking (%)	More than once a month	350 (15.2)	314 (15.1)	36 (16.2)	0.878
	Less than once a month	149 (6.5)	134 (6.4)	15 (6.8)	
	Never	1806 (78.4)	1635 (78.5)	171 (77.0)	
Physical Activity (%)	Yes	1611 (69.9)	1446 (69.4)	165 (74.3)	0.151
	No	694 (30.1)	637 (30.6)	57 (25.7)	
Internet Use (%)	Yes	199 (8.6)	188 (9.0)	11 (5.0)	0.054
	No	2106 (91.4)	1895 (91.0)	211 (95.0)	
Insurance (%)	Yes	2129 (92.4)	1923 (92.3)	206 (92.8)	0.905
	No	176 (7.6)	160 (7.7)	16 (7.2)	
Income Source (%)	Industry	120 (9.6)	111 (9.6)	9 (9.8)	0.364
	Agriculture	974 (78.1)	898 (77.7)	76 (82.6)	
	Both	153 (12.3)	146 (12.6)	7 (7.6)	
Income State (%)	< 16,000	1419 (78.7)	1288 (78.8)	131 (78.0)	0.021
	16,000–40000	263 (14.6)	245 (15.0)	18 (10.7)	
	> 40,000	121 (6.7)	102 (6.2)	19 (11.3)	
Residence (%)	City or town center	441 (19.1)	386 (18.5)	55 (24.8)	0.004
	City or town fringe	223 (9.7)	193 (9.3)	30 (13.5)	
	Rural areas	1641 (71.2)	1504 (72.2)	137 (61.7)	
Living Arrangement (%)	with spouse and other family members	413 (17.9)	382 (18.3)	31 (14.0)	< 0.001
	with family members without spouse	327 (14.2)	289 (13.9)	38 (17.1)	
	with spouse only	1100 (47.7)	1043 (50.1)	57 (25.7)	
	Alone	465 (20.2)	369 (17.7)	96 (43.2)	
Chronic Disease (%)	Yes	1261 (54.7)	1140 (54.7)	121 (54.5)	1
	No	1044 (45.3)	943 (45.3)	101 (45.5)	
Function (%)	ADL^1^&IADL^2^ limitations	399 (32.3)	363 (31.7)	36 (38.7)	0.382
	ADL limitations	76 (6.1)	71 (6.2)	5 (5.4)	
	IADL limitations	762 (61.6)	710 (62.1)	52 (55.9)	
Health Satisfaction (%)	No	932 (56.0)	853 (56.8)	79 (48.5)	0.109
	Somewhat	560 (33.7)	494 (32.9)	66 (40.5)	
	Yes	172 (10.3)	154 (10.3)	18 (11.0)	
Depression (%)	Yes	1114 (56.4)	1009 (56.4)	105 (56.5)	1
	No	862 (43.6)	781 (43.6)	81 (43.5)	

1. ADL refers to Activity of Daily Living

2. IADL refers to Instrumental Activities of Daily Living

It has been shown that for data with sample number > 1000 and missing values below 90%, the multiple missing values imputation method can be used to accurately estimate the regression model [28]. Based on the statistical results of this study, variables with a missing rate of > 60% were excluded from further analysis to reduce potential imputation errors, while for variables with a missing rate of ≤ 60%, Using “sklearn.Impute.IterativeImputer” module of “BayesianRidge” method to fill the missing data. We used robust multiple imputation based on Bayesian Ridge regression to handle missing values for all covariates before building machine-learning models. To ensure strict data isolation, the Imputer performs Fit only on the training set and estimates missing data for 10 iterations, and then uses the obtained parameters to Transform the test set, so that the test set data information does not participate in the training process.

### Screening and construction of ML models

#### Feature selection via LASSO

To ensure model parsimony and mitigate the risk of overfitting caused by candidate variables, we performed feature selection on the initial predefined variables. A Least Absolute Shrinkage and Selection Operator (LASSO) logistic regression model with L1 regularization (penalty parameter C = 0.1) was fitted exclusively on the training set. LASSO effectively shrinks the coefficients of less contributory variables to exactly zero, thereby selecting only the most robust predictors for subsequent modeling.

#### Cost-sensitive learning

In this study, the dependent variable (care type) was dichotomous with a strong imbalance between the informal and formal care groups (90.4% vs. 9.6%, respectively), which may lead to serious decline in the classification performance of the ML model [29]. Instead of employing traditional synthetic data generation techniques (such as SMOTE) which may introduce excessive noise and degrade classification performance, we utilized a cost-sensitive learning approach. Specifically, optimal class weights were assigned algorithmically during model training. This method applies a higher concomitant loss penalty for the misclassification of the minority class (formal care), inversely proportional to their empirical frequencies in the training set. This algorithmic adjustment for class imbalance was natively implemented using identical parameters embedded in the core training modules of the respective machine learning libraries. By seamlessly integrating the loss-penalization within the native algorithmic architecture, this approach robustly corrects biases related to imbalanced data while strictly preserving the authenticity of the original demographic distribution. This algorithm has been widely applied in prediction models to correct biases of imbalanced data [30] [PMID: 39288404].

#### Generation of prediction models

The data were randomly allocated to the training or validation set. Different ML algorithms were employed using the training set to build the prediction models. The predictive accuracy of the ML models was assessed by comparing the dispersion between the predicted and actual values [31].

In this study, the dataset was initially partitioned via stratified random sampling into a training set (70%) and an independent test set (30%).To rigorously ensure no data leakage occurred during the preprocessing phase, all subsequent model training and hyperparameter tuning were conducted strictly within the training set, while the test set was uniquely reserved for final performance assessment.

Following feature selection and dealing with data imbalance, the data were analyzed utilizing 11 diverse ML algorithms, integrating linear/traditional models, tree-based ensemble models, and neural networks. These models included Logistic Regression, Gaussian Naive Bayes, K-Nearest Neighbor, Support Vector Machine, Multi-Layer Perceptron, Decision Tree, Random Forest, Gradient Boosting Machine, eXtreme Gradient Boosting (XGBoost), Light Gradient Boosting Machine (LGBM), and CatBoost. Incorporating ML models based on divergent mathematical principles allows us to transcend the limitations of single conventional models and comprehensively determine the most suitable prediction algorithm for long-term care needs in China.These models are available through the Python packages “sklearn” [32], “xgboost” [33, 34], “lightgbm” [35, 36], and “catboost” [37, 38].

In order to maximize predictive performance and avoid overfitting, hyperparameter optimization was rigorously conducted for all ML models. This was achieved using the GridSearchCV function from the Python package 'scikit-learn' [32], employing a fivefold cross-validation scheme exclusively within the training set.The final parameters are presented in Supplemental Material 2. After optimal parameter adjustment, the prediction efficiency was calculated. All 11 models were evaluated and the optimal models were selected for the best modeling.

#### K-fold cross validation

K-fold cross validation was used to evaluate the hyperparameter optimization and predictive performance of the ML models. In this process, the data were allocated to the K-1 group for use as the training data or the remaining group for use as validation data.

In this study, during the hyperparameter optimization process of the models, a fivefold cross-validation process was strictly nested within the training set to obtain optimal parameters. The algorithm was implemented with the Python package “sklearn” [32].

### Evaluation of predictive efficacy

Based on the results of K-Fold cross validation, five parameters, including accuracy, precision, recall, F1 score, and area under the receiver operating characteristic curve (AUC), were used to evaluate the ML models (Table 2), and the optimal algorithm was selected to build a prediction model.

**Table 2:** The performances of the 11 ML models in Training and Testing set

	Model	Accuracy	Precision	Recall	F1-score	AUC
Training set	CatBoost	0.7266	0.2082	0.6581	0.3163	0.7704
	DecisionTree	0.6348	0.1609	0.6645	0.2591	0.685
	GBM	0.9089	0.8333	0.0645	0.1198	0.7695
	GaussianNB	0.8791	0.2436	0.1226	0.1631	0.6897
	KNN	0.9039	0.500	0.0065	0.0127	0.7865
	LightGBM	0.7179	0.200	0.6452	0.3053	0.7702
	LogisticRegression	0.6764	0.1706	0.6129	0.2669	0.7118
	MLP	0.9089	0.900	0.0581	0.1091	0.7976
	RandomForest	0.7508	0.201	0.5355	0.2923	0.7284
	SVM	0.6782	0.1654	0.5806	0.2575	0.7054
	XGBoost	0.7192	0.2032	0.6581	0.3105	0.778
Testing set	CatBoost	0.6705	0.166	0.597	0.2597	0.6831
	DecisionTree	0.5968	0.1581	0.7313	0.2599	0.6807
	GBM	0.896	0	0	0	0.6776
	GaussianNB	0.8627	0.0625	0.0299	0.0404	0.6608
	KNN	0.9003	0	0	0	0.5958
	LightGBM	0.6705	0.1687	0.6119	0.2645	0.6935
	LogisticRegression	0.6474	0.171	0.6866	0.2738	0.682
	MLP	0.8974	0	0	0	0.6622
	RandomForest	0.724	0.1869	0.5522	0.2792	0.7014
	SVM	0.659	0.166	0.6269	0.2625	0.6855
	XGBoost	0.6749	0.1736	0.6269	0.2718	0.6678

*Abbreviations:* *GBM* Gradient Boosting Machine, *GaussianNB* Gaussian Naive Bayes, *KNN* K-nearest neighbor classification, *MLP* Multi-Layer Perceptron, *SVM* Support Vector Machine, *XGBoost* eXtreme Gradient Boosting

### Interpretability

The “black box” characteristic of ML algorithms may hinder building a prediction model, which complicates evaluation of ML models. In this study, we used the interpretability framework SHAP (SHapley Additive exPlanations) [39, 40], which offers extensive information as compared to other interpretability frameworks [41]. We should emphasize that SHAP values reflect feature contributions within the fitted machine learning model and represent model-based associations, they do not imply causality (Table 3).

**Table 3:** The SHAP value of the control variables in Random Forest model

Variables	Mean_Abs_(SHAP)
Marital Status	0.061099
Living Arrangement	0.047005
Social Activity	0.024145
Physical Activity	0.016743
age	0.014285
Residence	0.01052
Smoking Status	0.009685
Gender	0.007596
Online Communication	0.005107
Drinking Status	0.004618
Insurance Coverage	0.003602

Crucially, to prevent data leakage and ensure an unbiased estimation of feature contributions, all SHAP analyses were strictly calculated using the entirely unseen, independent test set array. The study focuses on long-term care utilization among Chinese older adults based on the Anderson model and uses the interpretability framework SHAP to accomplish the following four objectives:(1)Identify the most critical long-term care associated factors; (2) Quantify the importance of each control variable; (3) Explore the complex interaction of control variables under the Anderson model and (4) Analyze the specific roles of key control variables in the study based on the importance ranking of control variables.

## Results

### Descriptive study

Of the 2305 individuals included in this study (Table 1), 2083 (90.4%) received only informal care and 222 (9.6%) received formal or combined care. As shown in Table 1, individuals aged ≥ 80 years (*p* < 0.001), not married (*p* < 0.001), more socially active (*p* < 0.001), who never smoked (*p* = 0.004), with higher incomes (*p* = 0.021), city dwelling (*p* = 0.004), and living alone (*p* < 0.001) preferred to use formal care.

The Andersen healthcare utilization model showed that predisposing and enabling factors affected the type of LTC utilization of older adults needing care, while there was no difference in need factors.

### Predictive performance of the ML models

To effectively mitigate multicollinearity and high-dimensionality, LASSO regression was executed as a pre-modeling feature selection step. Out of the initial pool of variables, 11 robust predictors were ultimately retained for the construction of subsequent prediction models, including: gender, age, marital status, social activity participation, smoking status, drinking status, physical activity participation, internet use (online chat), insurance status, residence (location), and living arrangement. After variable selection, the number of events per variable (EPV) ratio in the training set increased from 9.12 to 14.09, which further enhanced the ability to prevent model overfitting.

**Fig. 2:**
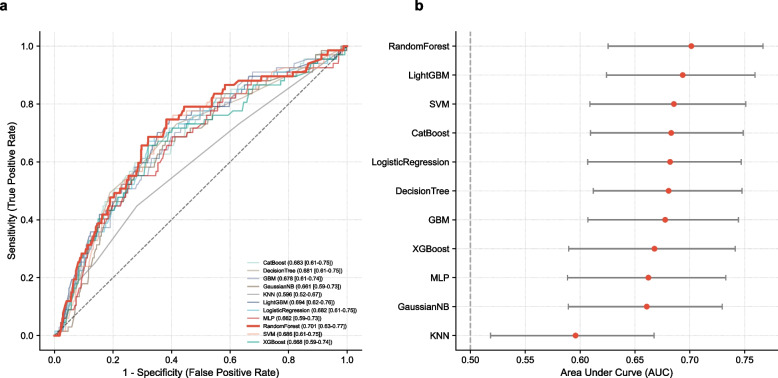
Performance

**Fig. 3:**
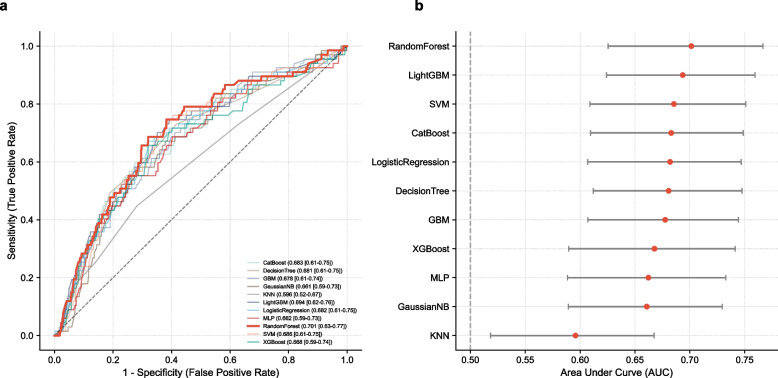
Clinical_Utility

The performances of the 11 ML models are shown in Table 2. Considering the strong imbalance of observed variables, the five parameters accuracy, precision, recall, F1 score and AUC score were included to minimize sample bias. Visualizations of the performance of the 11 ML models are shown in Figs. 2 and 3. Following comprehensive comparisons on the independent test set, the Random Forest model achieved the highest predictive performance, outperforming the underlying 10 algorithms with the most robust and optimal parameters (Supplemental Material 3). Additionally, the efficacy of the cost-sensitive learning approach was effectively validated; models parameterized with balanced class weights demonstrated significantly improved robustness towards identifying the minority class (formal care) compared to their unweighted generic counterparts (Figure S1). Furthermore, learning curve analyses confirmed that the optimized algorithms successfully converged with acceptable variance, mitigating concerns of substantial overfitting or underfitting (Figure S2). Because test set performance authentically reflects generalization robustness on new populations, the optimal Random Forest configuration was ultimately selected as the finalized prediction model for downstream demographic interpretation.

### Interpretability of the ML model

**Fig. 4:**
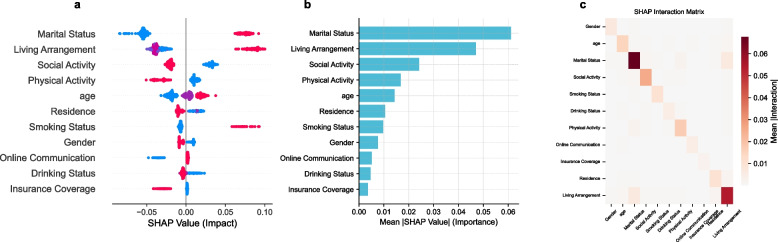
Interpretation

The Random Forest algorithm was used to construct the prediction model. The interpretability of the model is shown in Fig. 4. The potential impacts of the control variables on care type are shown in Fig. 4A. After the SHAP values were calculated, they were ranked from high to low according to the Feature values. A higher Feature Value represents a stronger effect of this variable on the type of care in the prediction model. A SHAP value > 0 indicated greater inclination to receive formal care. Red represents high formal care eigenvalues, blue represents low eigenvalues. Ranking the contribution of the control variables showed that marital status, living arrangement, social activity, physical activity, age and residence were the most significant control variables for formal care. Hierarchical clustering of the control variables showed similar results. As shown in Fig. 4B, the ranking map shows the mean SHAP value ranking in the study model. To further discuss the interaction between significant variables in the model, SHAP interactivity plots are plotted in Fig. 4C, which shows social activity, age, physical activity and residence were independent factors in the prediction model. However, marital status exhibited obvious interactions with living arrangement.

**Fig. 5:**
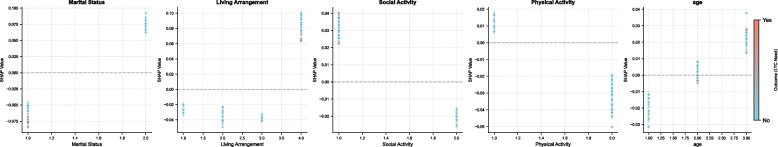
SHAP_Dependence_Top5

Next, the associated impacts of the top five control variables on the care type were determined. As shown in Fig. 5, the horizontal axis represents the coding corresponding to different assignments in the variable, the vertical axis is the SHAP value corresponding to the assignment, and SHAP value > 0 indicates a preference for formal care. Individuals who lived alone preferred formal care, while those who lived with a spouse only preferred informal care. Moreover, individuals who did not participate in social activities, over the age of 80, and participated in physical activities weekly were more inclined to use formal care. Thus, marital status, living arrangement and participation in social activities were identified as significant factors associated with the types of LTC utilization by older adults in China.

## Discussion

There has been no large-scale and quality-assured formal LTC system in China for a long time. Therefore, studies have mainly focused on informal LTC. However, with the aging population and low birthrate, the Chinese government began to actively build formal LTC programs in 2015. So, it is now possible to study the different types of LTC services used by older adults in China. To the best of our knowledge, this study is the first to explore such issues with the use of the latest national survey data.

According to the CHARLS 2020 data, 9.6% of older adults in China used formal care, although this proportion is very low as compared to some countries with complete LTC services. For example, 46% of older adults in Iceland reportedly only received formal care at home [6] and 53% of those in Belgium [16], as compared to only 1.92% in China in 2018, suggesting that the availability of formal care services should be increased. Hence, clarification of the different factors influencing the utilization of formal and informal care by older adults is useful to create LTC services in China [42, 43]. This type of research can be conducted in multiple fields, including politics, economics, and social studies [44], although the conclusions would vary depending on regions and cultures [45, 46]. Therefore, it is feasible, necessary, and interesting to identify the factors associated with utilization of different types of LTC by older adults in China. However, we should explicitly declare that the primary aim of this study is explanatory and exploratory, and it is not intended for direct clinical or policy deployment.

This study used the Andersen healthcare utilization model to analyze 18 factors influencing utilization of different types of LTC in China. Because the data analysis tool required the elimination of variables with missing values > 60%, we reluctantly removed the level of education in the subsequent analysis, while retaining the other 17 predisposing, enabling, and need factors. Here, conventional regression methods were not used for statistical analysis, but rather ML methods to identify the most important associated factors and avoid bias caused by unbalanced samples. ML methods have been widely used in medical research for drug development [47, 48], disease diagnosis and treatment [49, 50], and identification of high-risk populations [51]. However, ML methods are rarely used in LTC research.

This study used 11 ML methods, more than most previous studies. Comparisons of accuracy found that Random Forest had better performance with the training set. Therefore, the identified factors associated with the types of LTC utilization were the most reliable. The Random Forest model performed better in multiple evaluation indices in this study. It may be that the Random Forest model improves the robustness of the model in stepwise optimization, while sacrificing the training speed but maintaining high accuracy. This study explored the associated with factors of long-term care choice tendency under the background of Anderson model. The interaction between variables was strong and the relationship was complex. Compared with other models, the Random Forest model showed relatively better accuracy in identifying the main associated factors of long-term care utilization among Chinese older adults. However, given the highly imbalanced setting and the model’s overall performance, our result should be regarded as providing a directional indication rather than robust prediction. Meanwhile we should emphasize that although the ML model is used to “predict” the associated factors of long-term care, it is intended to “predict” the strength of observed associations within the data, not to infer causality. Our study also considered the interactions of multiple control variables, because it was not meaningful to discuss the influence of single factors. After combining the significant results of the chi-square test, marital status, living arrangement, and social activity were identified as the main associated factors of the types of LTC utilization by older adults in China.

Across our multiple analyses, living arrangement consistently emerged as one of the most significant and stable associations. Given its clear interactions with marital status, we consider living arrangement was the key associated factor. We found that older adults living alone were most likely to use formal care and least likely to use informal care, in agreement with studies conducted in Canada in North America and South Korea in East Asia. Older adults living alone had very few people available to provide informal care and, thus, had to choose formal care [52, 53]. In addition, many older adults in China live alone have no spouses or children, often leaving them trapped in poverty. While public support for LTC remains limited in China, local governments do provide some subsidies for impoverished older members in need of care [54]. This, to some extent, increases the likelihood that they may choose formal care services. On the contrary, older adults not living alone mostly lived with their spouses and children who could provide timely and adequate informal care [55, 56], which reduced formal care utilization [57].

In this ML analysis, marital status emerged as the most significant associations. Marital status is intrinsically linked to living arrangement. Older adults living with family members other than a spouse were more likely to use formal care than those living with family members and a spouse, while those living with a spouse were the least likely to use formal care and the most likely to use informal care. These phenomena indicate that a spouse was the most important provider of informal care, which was very similar to the situation in Nordic countries, even if the partners were not always in a marriage relationship [58]. This may be due to the fact that a partner in an intimate relationship was more likely to provide care [59], and the caregivers were mostly elderly partners who had more personal time than younger people [60]. This was a notable change in China, which traditionally has maintained a strong emphasis on filial piety. Hu et al. argued that the protective effect of a spouse providing informal care was not as strong as that provided by children in China [19], whereas the present study showed that a spousal caregiver contributed more informal care than children. However, the physical and mental burdens and health problems of spousal caregivers must also be considered [61, 62].

Social activity was another stable associated factor, which had been rarely addressed in previous studies of LTC in China. We found that only 32.6% of older adults with care dependency participated in social activities, but were much more likely to use formal care, while those who did not participate in social activities were slightly more likely to use informal care. This finding illustrates that participation in social activities could reflect social participation levels [63], as care dependency reduced social participation of older adults, which could increase the risk of chronic diseases [64, 65], further exacerbating functional decline [66], eventually increasing the burden of LTC systems. On the other hand, participation in social activities could promote access to formal care. This finding differs from a study by Stafford & Kuh, which found that limited social contact was associated with expecting formal care, because these older adults people had fewer relatives in social networks, resulting in the inability to use informal care [59]. However, this study only addressed care expectations, rather than actual utilization. Nonetheless, both studies agreed that even limited social participation could increase social support for older adults [67], thus increasing the possibility to use formal care [45].

In general, the enabling and predisposing factors ranked higher than the need factors. Some studies have reported that socioeconomic factors had a greater impact on the types of LTC utilization by older adults than health needs [68]. Greater availability of public funds increases availability and utilization of LTC services [69, 70]. The goal of LTC systems is to meet the care needs of older adults. Therefore, the Chinese government should attempt to improve the availability of LTC services even without a significant increase in public spending. In this study, we propose the following recommendations:

First, governmental LTC programs should address the burden and health of spousal caregivers, most of whom are also older adults. Existing research showed that the availability of care in China had little negative impact on the happiness and health of caregivers, although the high intensity of care remained quite significant [23, 71]. So, the family members of older adults with medium and severe function dependency should receive top priority for financial subsidies or free service, and community organizations should provide more assistance.

Second, the government and society should encourage and help older adults to participate in multiple social activities, especially those with functional disabilities. These measures include eliminating age and disability discrimination, strengthening age-appropriate social participation, and improving the ability of older adults to participate in social activities, as participation can improve the diversity of older adults using LTC, so as to better use LTC services relative to individual needs.

Third, the government should vigorously create formal care service systems in urban fringe areas, which would be easier than in rural areas because of the availability of technical guidance and financial support in urban areas. However, increased availability of LTC services would likely attract older adults living in surrounding rural areas to relocate to urban areas to use formal care services.

Several limitations to this study should be noted. First, the CHARLS database provides comprehensive care information in China. However, this database was last updated in 2020 for various reasons. Thus, a new wave of data is eagerly anticipated to illustrate changes to LTC services in China, especially regarding factors influencing utilization. Additionally, considering the vast regional differences across China, factors such as regional policy variation, unmeasured institutional supply factors (the availability and quality of local care institutions), and cultural heterogeneity, these unmeasured variables may potentially limit the generalizability of our findings to specific sub-populations or regions within the country. Second, home isolation and community lockdowns caused by COVID-19 in 2020 may have limited access of care previously available to older adults. Third, in this study, fewer older adults used formal care. The relatively modest sample size may introduce potential biases, thereby limiting the generalizability of our findings. Fourth, the integration of ML techniques within the Andersen model framework remains underdeveloped, posing challenges in optimally selecting ML algorithms for analysis, which may affect result accuracy. Fifth, this was a cross-sectional study, therefore, it was not possible to rule out the possibility of reverse causation, thereby warranting further research to investigate a potential causal mechanism. Similarly, SHAP values only explain feature contributions within the fitted model and do not represent causal determinants. Finally, we must admit that our model was developed and validated on a single, specific cohort. The absence of external validation in geographically or temporally distinct populations means that its transportability and performance over time remain uncertain.

## Conclusion

Using the latest wave of CHARLS data with the Andersen healthcare utilization model, we investigated the types of LTC utilization by older adults in China and identified the factors associated with the use of informal and formal care. ML models were used to rank the significant associated with factors, which found that living arrangement, social activity, and residence were the top ranked factors, while enabling and predisposing factors ranked higher than the need factors overall. Due to the impossibility of a rapid increase in fiscal expenditures for LTC services, the Chinese government should engage some special measures to make LTC utilization more fair and reasonable, such as lightening the burden of partner caregivers, increase participation of older adults in social activities, as well as strengthen formal care services in urban fringe areas.

## Supplementary Information

Supplementary Material 1.

Supplementary Material 2.

Supplementary Material 3.

Supplementary Material 4.

Supplementary Material 5.

## Data Availability

The data for this study were obtained from the CHARLS 2020, https://charls.pku.edu.cn/

## Abbreviations

LTC: Long-term care
ML: Machine learning
CHARLS: China Health and Retirement Longitudinal Study
SHAP: SHapley Additive exPlanations
