# Supplementary material for: Identifying the factors influencing long-term care utilization by older adults in China: machine learning analysis
Source: BMC Geriatr. 2026 May 20;26:953. doi: 10.1186/s12877-026-07652-y (PMC13366875; doi:10.1186/s12877-026-07652-y)
Supplement: Supplementary file 1 — Supplementary Material 1. [file 12877_2026_7652_MOESM1_ESM.docx]

**Predisposing factors：**

(1) Gender：xrgender, male=1 female=2

(2) Age: xrage, re-graded according to age, 60 ≤ age < 70 = 1,70 ≤ age < 80 = 2, age ≥ 80 = 3

(3) Education: Based on the question ba010

| Code | Raw answers |
| --- | --- |
| Below primary school (including private school)=1 | 1. Uneducated (illiterate)  2. Did not finish elementary school  3. Graduated from private school |
| Primary school=2 | 4.Elementary school graduation |
| Junior high school=3 | 5. Junior high school graduation |
| Senior high school and above=4 | 6. Graduation from high school  7. Graduation from junior colleges (including secondary teachers' colleges and vocational colleges)  8. College graduation  9. Bachelor's degree  10. Master's degree  11. graduation of doctoral degree |

(4) Marriage: Based on the question ba011

| Code | Raw answers |
| --- | --- |
| Yes=1 | 1.Married and living with spouse  2.Married, but not living with spouse temporarily due to work, etc.  3.Separated (no longer living together as spouses) |
| No=2 | 4. Divorced  5. Widowed  6. Never married |

(5) Social activity: Based on the question da038

| Code | Raw answers |
| --- | --- |
| Yes=1 | 1. Visiting and socializing with friends  2. Playing mahjong, chess, cards, going to the community room  3. Offer help to family members, friends or neighbors with whom you do not live  4. Dancing, working out, practicing qigong, etc.  5. Participate in community organization activities  6. Volunteering, or charitable activities, or caring for sick or disabled people who do not live with you  7. Attending school or training courses  8. Other social activities |
| No =2 | None of the above |

(6) Smoking: Based on question da047

| Code | Raw answers |
| --- | --- |
| Have a smoking habit=1 | 1. Still smoking  2. Quit smoking |
| Never=2 | 3. Never smoked |

(7) Drinking: Based on the question da051, original answer retained: More than once a month=1; Less than once a month=2; Never=3

(8) Physical activity: Based on the three questions attached to the question da032, as long as one of the answers is “Yes”, it is considered to be participating in sports activities, otherwise it is not participating in sports activities, and the final assignment is Yes=1, No=2.

(9)Internet Use：Based on the question da040, original answer retained, Yes=1, No=2

**Enabling factors：**

(1)Insurance：Based on the question ba016

| Code | Raw answers |
| --- | --- |
| Yes=1 | 1. Annual contribution (BA016_1) yuan  2. Has health insurance but does not pay a contribution |
| No=2 | 3. Does not have any health insurance |

(2) Income source: In this section, subjects were categorized into three main groups according to the source of household income; industry was defined as those whose sources of income were wages, pensions and running a business; the agriculture was defined as those who worked in planting, forestry and animal husbandry; and those who had both types of income were defined as another new group; the sources of the specific questions are reflected in the table.

| Code | Raw answers |
| --- | --- |
| Industry =1 | Answer “yes” to any of the options in gd001(Self-employment or starting a private business),ga001(Acceptance of wages) is considered to be consistent with the characteristics of the industrial population. |
| Agriculture =2 | Answer “yes” to any of the options in gc001(Agriculture),gc003(planting or forestry).gc005(Pastoralism or aquaculture) is considered to be consistent with the characteristics of the agricultural population. |
| Both=3 | Both incomes include |

(3)Income state：Individual income was graded based on two sources: salary (ga002) and pension (ga005).For respondents who answered a general range of income rather than a specific number, we used averaging to determine their specific income. After completing the income summary, the income was graded:<16,000=1;16,000-40,000=2>40,000=3

(4) Residence: Based on the question ba008

| Code | Raw answers |
| --- | --- |
| City or town center=1 | 1. City or town center area |
| City or town fringe =2 | 2. urban-rural or town- rural fringe area |
| Rural areas=3 | 3. Rural areas  4. special areas (pastoral, forest, etc.) |

(5) Living arrangement: This variable was based on the question ba011 and the 14 sub-questions of ca014. Married and currently cohabiting are considered to be living with their spouse; while other household members were determined based on the sub-questions of ca014 and were defined as living together when the household member has accompanied the respondent for >6 months.

| Code | Raw answers |
| --- | --- |
| with spouse and other family members =1 | 1. Living with spouse &  2.Living with more than one family member |
| with family members without spouse =2 | 1.Living without spouse &  2.Living with more than one family member |
| with spouse only =3 | 1.Living with spouse &  2.Not living with family members |
| Alone=4 | None of the above |

**Need factors：**

(1)Chronic Disease：Based on the question da003 with 15 sub-questions, as long as the answer to one of the questions was “Yes”, it was considered to have a chronic disease, and finally assigned a dichotomous variable, Yes=1, No=2.

(2)Function：

Based on the answers related to ADLs and IADLs questions.

①ADL: dressing(db001), bathing(db003), eating(db005), getting up(db007) and toileting(db009)

②IADL: housework(db012), cooking(db014), shopping(db016), phone calls(db018), take medication (db020), managing money (db022).

③Respondents who answered one or more of the following questions: “Difficult, need help” or “Unable to complete” were considered to have ADL or IADL limitation, and it was a dichotomous variable, yes = 1, no = 0.

④Constructed variables based on ADL and IADL responses: Function

| Code | Raw answers |
| --- | --- |
| ADL&IADL limitations=1 | The defects of both ADL and IADL function |
| ADL limitations=2 | The defects of ADL function |
| IADL limitations=3 | The defects of IADL function |

(3) Health Satisfaction: Based on question da001

| Code | Raw answers |
| --- | --- |
| No =1 | 4. Not good  5. Very bad |
| Somewhat=2 | 3. Average |
| Yes=3 | 1. Very good  2. Good |

(4) Depression: Based on the question dc016-dc025

The depression was measured by the 10-item Brief Depression Scale of the Self-Rating Scale of Depression (CES-D) (score ranging 0-30), and the score >=10 was recognized as depressive symptoms. The depression was a dichotomous variable, yes = 1, no = 0.
