# Supplementary figures and images for: Identifying the factors influencing long-term care utilization by older adults in China: machine learning analysis
Source: BMC Geriatr. 2026 May 20;26:953. doi: 10.1186/s12877-026-07652-y (PMC13366875; doi:10.1186/s12877-026-07652-y)

Impact of Class Weight Adjustment on Model Performance

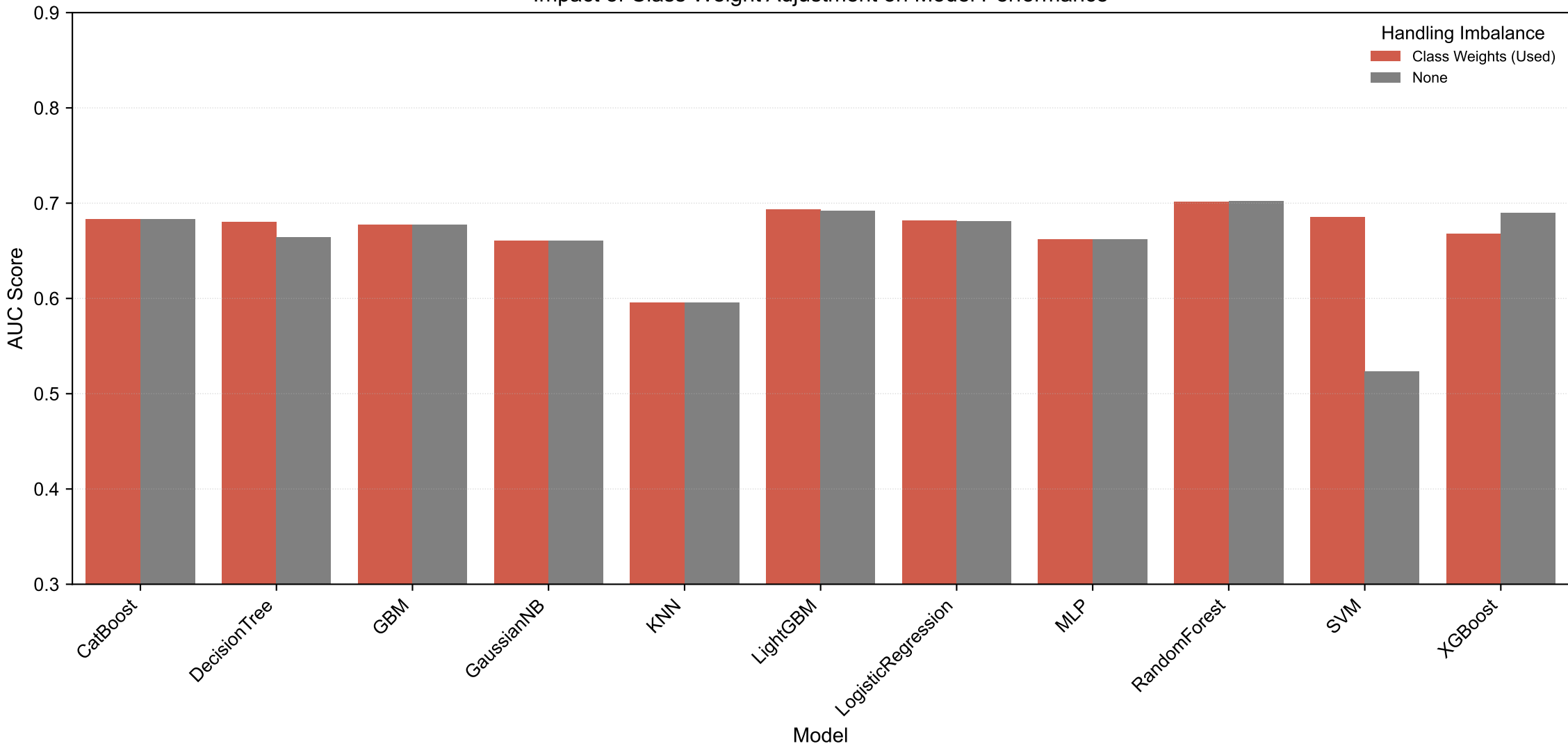

Supplement: Supplementary file 4 — Supplementary Material 4. [file 12877_2026_7652_MOESM4_ESM.pdf]

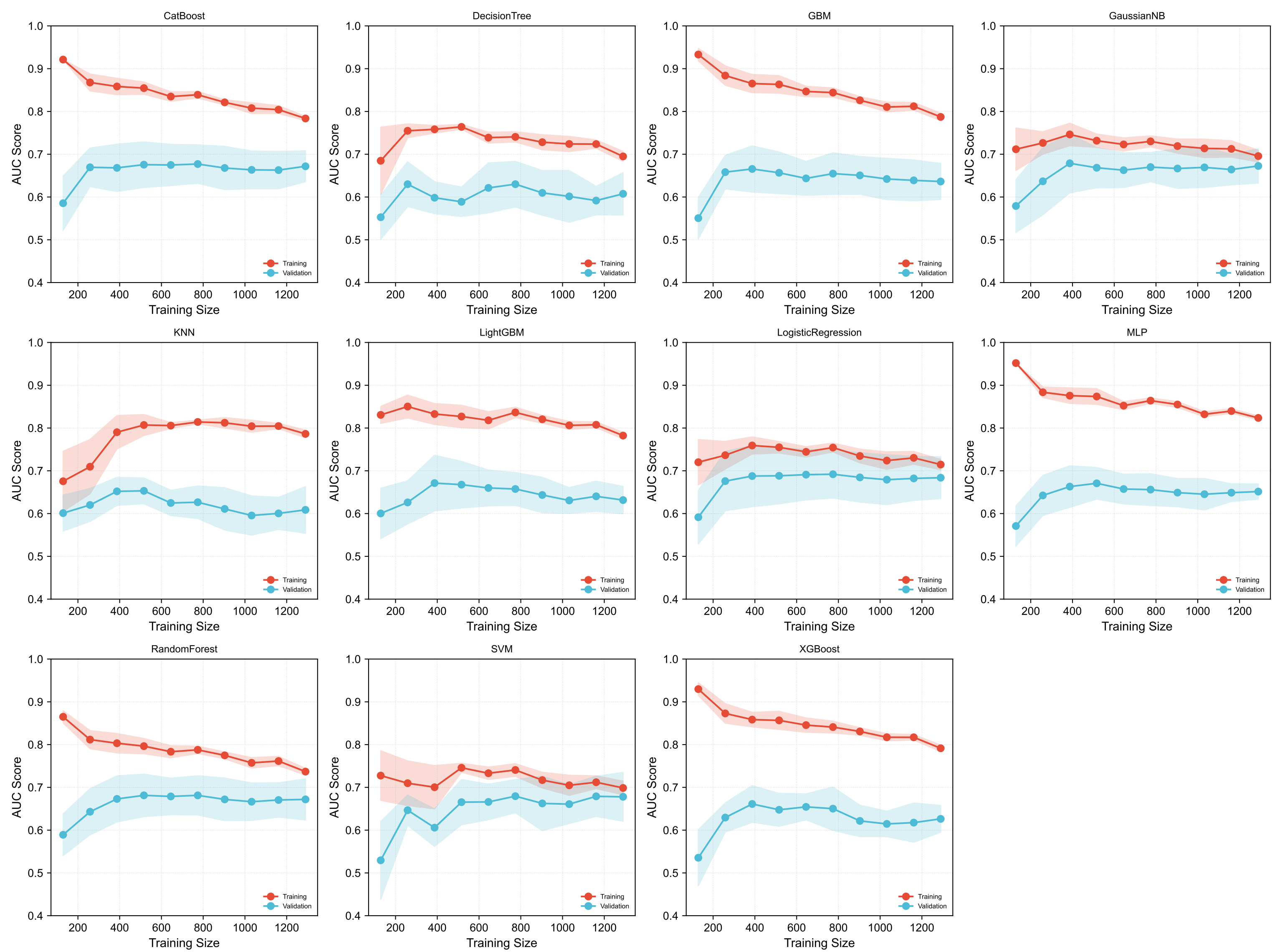

Supplement: Supplementary file 5 — Supplementary Material 5. [file 12877_2026_7652_MOESM5_ESM.pdf]
